# Psilocybin and Chronic Pain: A New Perspective for Future Pain Therapists?

**DOI:** 10.3390/medsci13040277

**Published:** 2025-11-20

**Authors:** Silvia Natoli, Arturo Cuomo, Maurizio Marchesini, Livio Luongo, Giuliano Lo Bianco, Vittorio Andrea Guardamagna, Shigeki Yamaguchi

**Affiliations:** 1 Department of Clinical-Surgical Diagnostic and Pediatric Sciences, University of Pavia, 27100 Pavia, Italy; silvia.natoli@unipv.it; 2 Unit of Pain Therapy Service, Foundation Istituto di Ricovero e Cura a Carattere Scientifico (IRCCS), Policlinico San Matteo, 27100 Pavia, Italy; 3 Division of Anesthesia and Pain Medicine, Istituto Nazionale Tumori, IRCCS-Fondazione G. Pascale, 80131 Naples, Italy; a.cuomo@istitutotumori.na.it; 4 Department of Experimental Medicine, Pharmacology Division, University of Campania “L. Vanvitelli”, 80138 Naples, Italy; livio.luongo@unicampania.it; 5 Anesthesiology and Pain Department, Foundation G. Giglio Cefalù, 90015 Cefalù, Italy; giulianolobianco@gmail.com; 6 Department of Anesthesia, European Institute of Oncology (IEO), 20141 Milan, Italy; vittorio.guardamagna@ieo.it; 7 Department of Anesthesia and Pain Medicine, Dokkyo Medical University School of Medicine, Tochigi 321-0293, Japan; shigeki@dokkyomed.ac.jp

**Keywords:** psilocybin, chronic pain, neuronal plasticity, pain management, hallucinogens, neuroinflammation

## Abstract

Background: Chronic pain affects nearly one in five adults worldwide and remains a major healthcare burden due to its persistence, multidimensional impact, and resistance to conventional therapies. The opioid crisis has further highlighted the urgent need for safer and more effective alternatives. Psilocybin, a serotonergic psychedelic compound, has re-emerged as a potential therapeutic option for chronic pain given its effects on neuroplasticity, neuroinflammation, and emotional regulation. Methods: This narrative review synthesized evidence from published preclinical and clinical studies. The focus was on the mechanisms of action of psilocybin, animal models of neuropathic and inflammatory pain, and early human trials exploring its effects on pain, mood, and quality of life. Results: Preclinical studies demonstrated that psilocybin promotes synaptogenesis via BDNF-TrkB signalling, modulates 5-HT2A receptor activity, and reduces neuroinflammatory processes, leading to persistent analgesic and anxiolytic effects. Animal models of chemotherapy-induced neuropathy and inflammatory pain showed long-lasting antinociceptive responses. Clinical studies, though limited, reported improvements in depression, anxiety, resilience, and quality of life in patients with advanced cancer and chronic conditions, with preliminary evidence of analgesic benefit. Conclusions: Psilocybin shows promise as a multidimensional therapy for chronic pain, addressing both sensory and affective components. However, ethical issues, safety concerns, and regulatory barriers necessitate careful management, and robust randomized controlled trials are essential to confirm efficacy and guide clinical translation.

## 1. Introduction

Chronic pain, characterized by its persistence [1] and resistance to traditional treatments, represents a critical challenge for healthcare systems. Its substantial prevalence, roughly 20% of the population in Western countries [2], has led to heavy social and economic burdens, exacerbated by the inadequacy of current pharmacological interventions for many patients [3]. In light of the opioid epidemic, the need for alternative pain management strategies has grown increasingly urgent [4]. The pursuit of innovative and effective treatments for chronic pain has led to the exploration of unconventional therapeutic avenues [5,6]. Among these, psychedelic drugs, once marginalized in scientific research due to their controversial cultural history, are now garnering renewed attention as potential candidates in pain management [7]. Psychedelic substances such as lysergic acid diethylamide (LSD), *N*,*N*-dimethyltryptamine (DMT)—the active part of ayahuasca—mescaline, and psilocybin have been researched for a long time because of their mind-altering effects, including their use in rituals [8].

Recent evidence suggests that some of these compounds might offer unique benefits by targeting the multidimensional nature of pain, addressing both the physical and psychological components [9]. Indeed, emotional processes are now seen as important factors in causing and sustaining chronic pain [10], and mechanisms such as neuroplasticity in pain processing networks and neuroinflammation seem to be crucial to the maintenance of chronic pain states. Thus, psychedelic drugs, by affecting neuroplasticity and neural networks [11] and by modulating inflammation and the neuroimmune axis [12], present promising therapeutic opportunities.

As research momentum has accelerated over the last decade, increasing numbers of clinical trials have illuminated the potential of psychedelics to address conditions such as cancer-related pain, fibromyalgia, and phantom limb pain [13]. Despite regulatory barriers and methodological constraints, current findings appear promising for future investigation and clinical integration. In particular, psilocybin, [3-[2-(dimethylamino)ethyl]-1H-indol-4-yl] dihydrogen phosphate, similar to ketamine, has shown sustained antidepressant effects 1 week after assumption that last for up to 12 months [14,15], without serious adverse effects [16]. The US Food and Drug Administration has given psilocybin a breakthrough therapy designation for major depressive disorder, and the European Medicines Agency has approved psilocybin for use in phase III clinical studies of treatment-resistant depression [17].

This work employs a narrative methodology to discuss the therapeutic properties of the psychedelic psilocybin, its mechanisms of action, and the ethical considerations involved in its application by examining preclinical and clinical studies.

## 2. Materials and Methods

A comprehensive literature search was conducted in PubMed/MEDLINE, Google Scholar, and Web of Science, covering the period from January 2000 to March 2025. Only studies published in English were considered.

Two independent reviewers (M.M. and S.N.) screened the titles and abstracts of clinical studies, while preclinical studies were reviewed and selected by L.L. Disagreements were resolved by consensus after full-text evaluation. Systematic reviews and meta-analyses were examined to ensure completeness of the search and used as secondary references.

### Search Strategy

For preclinical studies, the following Boolean string was applied (with appropriate adaptation across databases):

“psilocybin” AND (“pain” OR “nociception” OR “neuropathic” OR “inflammatory” OR “mechanism” OR “neuroplasticity” OR “neuroinflammation”).

For clinical studies, the following Boolean string was applied:

“psilocybin” AND (“chronic pain” OR “neuropathic pain” OR “nociplastic pain” OR “headache” OR “migraine” OR “cluster headache” OR “fibromyalgia” OR “complex regional pain syndrome”).

Preclinical studies were included if they investigated the effects of psilocybin (or its active metabolite psilocin) on experimental models of nociception or neuropathic or inflammatory pain, or if they examined relevant mechanistic pathways such as serotonergic signalling, brain-derived neurotrophic factor (BDNF)-TrkB activation, or modulation of neuroinflammation. Mechanistic studies without direct pain outcomes were considered if they provided insight into neurobiological processes relevant to pain chronification.

Clinical studies were included if psilocybin was administered to human subjects with chronic pain conditions, including headache disorders, neuropathic pain, fibromyalgia, and complex regional pain syndrome (CRPS). Eligible designs comprised randomized controlled trials, open-label pilot studies, case series, and single-patient case reports. Outcomes of interest were changes in pain intensity or frequency, pain interference with daily life, quality of life measures, psychological comorbidities (e.g., depression, anxiety), and safety or tolerability.

The complete set of database-specific search queries, with Boolean operators, field tags, controlled vocabulary (MeSH/Emtree), and all applied limits, is presented in the Supplementary Materials (Table S1).

## 3. Discussion

### 3.1. Preclinical Evidence of Psilocybin in Pain Management

Psilocybin, [3-[2-(dimethylamino)ethyl]-1H-indol-4-yl] dihydrogen phosphate, is the main psychoactive component of psychedelic mushrooms. It has shown quick effects on synapse growth in early studies, marked by total number of dendritic branches and dendritic spine density in the prefrontal cortex (PFC) and hippocampus. These effects have been linked to antidepressant-like effects in mice [18]. Neuroplasticity promotion and plasticity-related behavioural effects are exerted through psylocibin’s high-affinity binding to TrkB. Of note, psychedelics act allosterically on TrkB by facilitating the effects of endogenous BDNF [19], the TrKB ligand [20]. Since BDNF is released in stimulated synapses, psilocybin promotes and strengthens activity-dependent plasticity by selectively stabilizing active synapses at the expense of inactive ones.

Insights into pain mechanisms have revealed that the pain experience is built in the cortex from nociceptive information and that circuits involved in cognitive and emotional processing are altered by chronic pain [21]. Particularly, researchers suggest that synaptic changes occurring in the anterior cingulate cortex (ACC) and in its connections with thalamic nuclei and medial prefrontal cortical regions are key features in chronic pain. They include potentiated excitatory synaptic transmission in the ACC [22] and increased strength of thalamus-to-ACC synaptic connections [23]. As a promoter of synaptic plasticity, BDNF appears to be a relevant actor in pain chronification, not only in neurons of the dorsal horn of the spinal cord, but also in supra-spinal areas.

Because a thorough report on BDNF and pain is beyond the scope of this review, we suggest M. Mazzitelli et al. for a more informative reading [24]. In brief, increasing evidence suggests that BDNF-TrkB signalling is fundamental for LTP and pain-related synaptic plasticity underlying pathological pain [25] in key areas involved in pain processing such as the spinal dorsal horn [26,27], nucleus accumbens (NAc) [28], medial prefrontal cortex (mPFC) [29], and ACC [30]. BDNF has complex and sometimes different effects in the spinal cord, the brainstem, and sub-cortical areas, where it has prevalent pronociceptive properties. In cortical areas, the picture is less clear, with BDNF in the ACC having facilitatory effects and positive effects on anxiety- and depression-like behaviours, whereas the effects of BDNF in the mPFC are still debated. Interestingly, enhancing pro-BDNF signalling in the ACC reduced chronic unpredictable mild stress (CUMS)-induced anxiety- and depression-like behaviours [31], revealing a unique role of BDNF in emotional–affective behaviours.

**Figure 1:**
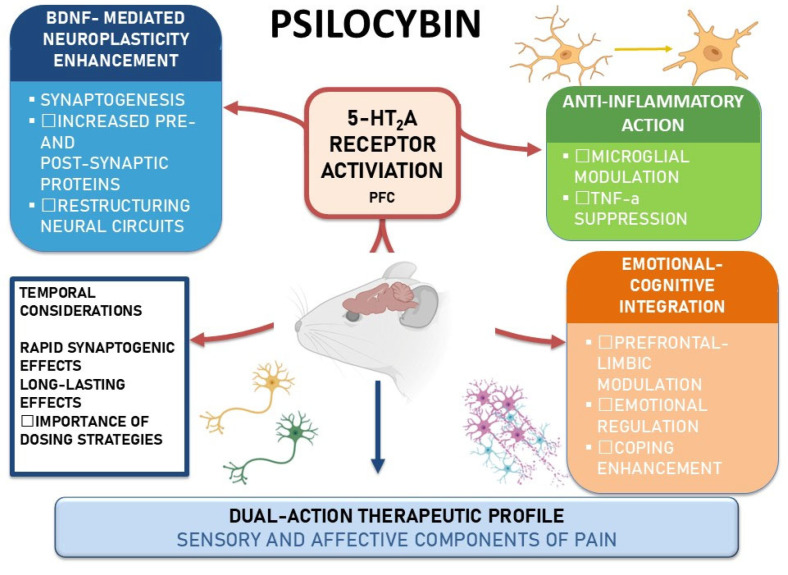
Psilocybin mechanisms in rodent models of pain. Schematic highlighting 5-HT2A receptor activation in the prefrontal/anterior cingulate cortex, with three convergent actions: BDNF-mediated neuroplasticity, anti-inflammatory effects, and emotional–cognitive integration. Temporal notes suggest a rapid onset with durable effects, supporting a dual-action profile on sensory and affective dimensions of pain. Abbreviations: BDNF, brain-derived neurotrophic factor; PFC, prefrontal cortex; TNF-α, tumor necrosis factor-alpha.

The mechanisms of action described in the preclinical setting are summarized in Figure 1.

#### 3.1.1. BDNF-Mediated Neuroplasticity Enhancement

Given the effects of psilocybin on the BDNF-TrkB pathway in cortical areas, it appears a promising candidate to exert pain-relieving effects by addressing not only the nociceptive components of pain but also its affective and psychological dimensions. Results from preclinical studies reveal that psilocybin can reverse anxiety-like behaviour in a mouse model of chemotherapy-induced peripheral neuropathy [32]. Several studies point out that changes in pain behaviour are the expression of synaptogenic changes induced by psilocybin, which may contribute to the restructuring of maladaptive neural circuits implicated in chronic pain, thus addressing both the sensory and emotional dimensions, reducing pain-related emotional distress, and improving coping mechanisms. Indeed, psilocybin can recalibrate activity in prefrontal and limbic regions, including the amygdala [33].

#### 3.1.2. Emotional–Cognitive Integration

It is well known that the 5-HT system plays a key role in descending pain facilitation or inhibition, and pain chronification may affect descending inhibitory serotoninergic pathways [34]. 5-HT2A receptor is highly expressed in areas involved in pain processing, including the frontal cortex, claustrum, and dorsal horn of the spinal cord. Moreover, it is known that 5-HT2A receptor activity modulates the neural circuits involved in integrating emotional and cognitive responses to pain, thereby influencing both pain perception and its accompanying psychological burden [33]. Therefore, this receptor may play a pivotal role in modulating pain perception and processing, offering a targeted approach that may reduce common adverse effects seen with non-specific analgesics [32].

Psilocybin has shown to reverse pain-like evoked behaviours via the 5-HT2A receptor [32] in two distinct models of chronic pain, namely chemotherapy-induced peripheral neuropathy (CIPN, neuropathic pain) and Complete Freud’s Adjuvant injection (CFA, inflammatory pain). It is believed that the effects of psilocybin on the serotoninergic system are mediated by psilocin, the active metabolite of psilocybin [35], which displays high-affinity agonism for the 5-HT7, 5-HT1D, and 5-HT2A, B, and C receptors [36]. Interestingly, psilocybin demonstrated antinociceptive effects that outlasted the half-life of psilocin [37]. Moreover, opposite to other psychedelics, psilocybin exerts long-lasting pain relief in neuropathic as well as inflammatory models of pain [32,38], suggesting more complex mechanisms and interactions and possibly changes in neural plasticity in the peripheral and/or central nervous system.

In spared nerve injury (neuropathic) and high-volume CFA (inflammatory) mouse models [39], a single 0.5 mg/kg dose of psilocybin rapidly reversed established mechanical allodynia within 24 h, and the effect persisted for at least 12 days, accompanied by parallel improvements in anxiety–depressive-like behaviors in both sexes. Mechanistically, analgesia was localized to the anterior cingulate cortex (ACC). Local psilocin in the ACC replicated behavioral benefits and suppressed pyramidal hyperactivity, whereas intrathecal delivery was ineffective, implicating a supraspinal, ACC-centric mechanism. Moreover, blocking either 5-HT2A or 5-HT1A signaling abolished both rapid and durable effects.

However, the long-term implications of modulating this receptor on neural circuits remain insufficiently studied. In addition, translating these preclinical findings into human treatment protocols involves addressing challenges such as receptor heterogeneity in human populations and the potential for variability in 5-HT2A receptor sensitivity.

This possible dual-action property places psilocybin as a unique therapeutic agent, especially when considering the psychological burden accompanying chronic pain. However, it remains unclear whether the observed synaptic remodelling is sufficient to produce clinically significant outcomes in diverse pain conditions, and possible other mechanisms may be involved in its net clinical effect. For example, it has been shown that in serotonin (5-HT)-depleted animals, suppression of BDNF-TrkB signalling in the RVM has antinociceptive, rather than pronociceptive, effects [40], suggesting an intricate relationship between the 5-HT system and BDNF signalling in pain processing.

#### 3.1.3. Anti-Inflammatory Action

Another possible mechanism by which psilocybin may provide pain relief is related to its possible effects on neuroinflammation. Again, the effects of psylocibin on BDNF-TrKA signalling appear to be of major relevance. Indeed, robust evidence highlights as BDNF is involved in the genesis of neuroimmune-maintained maladaptive plasticity in chronic pain. BDNF can be secreted by microglia upon several stimuli. For example, BDNF is secreted by activated microglia after nerve injury in male rats [41], ultimately disinhibiting GABA and glycine transmission and thereby enhancing spinal cord excitability. Intrathecal BDNF promotes microglia and astrocyte release of pro-inflammatory cytokines in the spinal cord, promoting neuroinflammation-related chronic pain states [42]. In microglial cells exposed to lipopolysaccharides (LPS), psilocybin and psilocin significantly suppressed TNF-α expression and increased BDNF levels, underscoring psilocybin’s immunomodulatory potential and supporting its therapeutic relevance in pain conditions characterized by immune dysregulation, such as neuropathic, inflammatory, and nociplastic pain [43]. Confirming the complex relationship with the serotoninergic system, experimental evidence suggests that the anti-inflammatory properties of psilocybin are mediated by 5-HT2A, 5-HT2B, 5-HT7, and TrkB signalling. However, how psilocybin induces changes in inflammatory mediators is still vague, and new pathways and mechanisms are continually added to our knowledge. For instance, AhR (Aryl hydrocarbon Receptor) activation was required for psilocin-induced BDNF upregulation but not TNF-α suppression [44].

By targeting inflammation at the microglial level, psilocybin introduces an innovative therapeutic mechanism. These benefits could expand its application beyond immediate analgesia to include neuroprotective strategies, particularly in progressive conditions such as neuropathic pain syndromes or degenerative disorders [43]. However, the broader systemic impact of psilocybin’s immunomodulatory effects remains poorly understood. Examining whether these effects could inadvertently suppress protective immune responses or whether long-term use could lead to tolerance or diminished efficacy is critical for advancing its clinical application.

Overall, preclinical findings strongly support the potential of psilocybin in chronic pain management by demonstrating their unique capacity to modulate both neuroplasticity and inflammatory pathways. Their multidimensional pharmacological profile addresses the multidimensional aspects of chronic pain, differentiating psychedelics from traditional analgesics that often fail to consider the broader impact of pain on patients’ lives.

### 3.2. Clinical Studies

The surge in registered clinical trials on psychedelic compounds since 2017, with 77.1% initiated since then, reflects the growing interest and investment in research in this area across various conditions, including chronic pain [45]. In particular, psylocibin garnered attention, echoing its potential in addressing both psychological and physical dimensions of pain [45].

A recent meta-analysis of randomized clinical trials has revealed that psilocybin combined with psychotherapy was more effective in relieving depressive symptoms compared to the comparator arms in patients diagnosed with life-threatening illnesses or major depressive disorder [46]. Across the included studies, psilocybin appeared to be well tolerated, with serious adverse events being rare [46].

Psilocybin has shown considerable long-lasting psychological benefits in ameliorating depression and anxiety in individuals facing fatal cancer [47]. Results were obtained when psilocybin was administered in hallucinogenic doses and coupled with psychotherapy. In this randomized controlled study on 51 patients with advanced cancer, high doses of psilocybin (22 or 30 mg/70 kg) were associated with sustained improvements in mood, quality of life, and optimism over a six-month period, outperforming very low placebo-like doses (1 or 3 mg/70 kg) [47]. Interestingly, a high percentage of participants (94%) identified their psilocybin treatment as among the most meaningful experiences of their lives. These results suggest that psilocybin may have an advantage over traditional pain relievers, addressing broader issues of emotional resilience and psychological well-being in patients with chronic conditions [48]. Indeed, its potential use in terminal illnesses demonstrates its capability to foster psychological strength, which may enhance patients’ quality of life and resilience against chronic pain [49]. Hence, by promoting a framework of psychological resilience, psilocybin shifts the focus of pain management from purely palliative care to a model that empowers patients to actively engage with their experiences.

Despite the importance of addressing psychological factors to improve pain coping strategies, clinical cases of patients affected by neuropathic pain show that psilocybin at low sub-psychedelic doses has purely analgesic effects [50].

Beyond chronic pain syndromes, psilocybin has also been investigated in primary headache disorders, where unmet therapeutic needs remain particularly pronounced. An exploratory double-blind, placebo-controlled crossover trial in patients with migraine demonstrated that a single low oral dose of psilocybin (0.143 mg/kg) in two sessions separated by two weeks significantly reduced weekly migraine days compared with placebo during the two weeks following administration (−1.65 vs. −0.15 days/week, *p* = 0.003) [51]. Psilocybin also led to greater reductions in migraine attack frequency, pain severity, functional impairment, and days of abortive medication use. Interestingly, these therapeutic effects were not correlated with the intensity of acute psychedelic experiences, suggesting that the benefits might be independent of hallucinogenic effect.

A patient-informed “pulse regimen” of psilocybin has been investigated in cluster headache, a condition with very limited effective preventive options. The first randomized, double-blind, placebo-controlled trial [52] evaluated three administrations of psilocybin (0.143 mg/kg, ~10 mg/70 kg) given five days apart, compared with a placebo. Sixteen participants were randomized, and fourteen were included in the final analysis. In the three weeks after treatment initiation, psilocybin reduced weekly attack frequency by about three attacks compared with baseline, whereas the placebo group showed no meaningful change. The difference did not reach statistical significance, though the effect size was moderate overall and larger in chronic patients, in whom reductions were maintained over eight weeks. A blinded extension phase was conducted [53], in which ten participants from the first study received a second psilocybin pulse at least six months later. In this setting, psilocybin produced a significant reduction in weekly attack frequency, from an average of 18.4 to 9.8 attacks per week (*p* = 0.013), corresponding to an approximately 50% decrease, irrespective of whether participants had responded during the first round. Reductions in attack severity and abortive medication use were also observed. A more recent phase Ib open-label study investigated ascending doses of psilocybin in patients with short-lasting unilateral neuralgiform headache (SUNHA) attacks [54]. Although the trial was limited by early termination and very small numbers, preliminary observations suggested a meaningful reduction in daily attack frequency in two out of three completers, accompanied by patient-reported changes in their relationship to pain. No major safety concerns emerged.

Across all clinical studies conducted to date, adverse events were few or absent, and when present they were mild and transient, never requiring treatment discontinuation.

Taken together, these early clinical findings across migraine, cluster headache, and SUNHA provide encouraging signals that psilocybin may exert therapeutic effects in primary headache disorders, potentially reducing attack frequency and improving patients’ ability to cope with their condition. Nevertheless, the evidence remains preliminary, derived from small pilot samples with limited follow-up.

A recent open-label pilot trial at the University of Michigan evaluated psilocybin-assisted therapy in five adults with fibromyalgia [13]. Participants received two oral doses of psilocybin (15 mg and 25 mg) in combination with structured psychotherapy. The treatment was well tolerated, with only mild and transient adverse events such as headache or gastrointestinal discomfort, and no serious complications. Clinically meaningful improvements were observed in pain severity, pain interference, and sleep disturbance, while some participants also reported better global functioning and psychological well-being. Although limited by small sample size and lack of controls, the findings provide preliminary support for the feasibility and potential benefit of psilocybin-assisted therapy in this nociplastic pain condition.

A recent case report described the experience of a 54-year-old woman with long-standing, treatment-refractory complex regional pain syndrome (CRPS) [55]. After multiple failed conventional therapies, including neuromodulation and ketamine, she underwent guided psilocybin sessions with adjunctive reprocessing techniques. Following three administrations of psilocybin mushrooms, her pain scores fell dramatically to near zero and remained stable for nine months. Although anecdotal, this case highlights psilocybin’s potential to provide sustained relief in otherwise refractory CRPS.

We identified eight clinical studies assessing psilocybin in pain conditions. Study characteristics and primary outcomes are summarized in Table 1.

Overall, these findings highlight the multifaceted therapeutic potential of psilocybin, which could be leveraged to create more comprehensive treatment protocols for those living with chronic pain. Positive results on major depression and anxiety were obtained in the context of supportive psychotherapy. It should be clarified whether the unique psychological mechanisms of psilocybin-assisted psychotherapy, which induces ego dissolution and promotes mindfulness-related cognitive processes [56], has a distinctive role that distinguishes it from traditional pharmacological treatments. However, critical questions remain regarding how these subjective psychological effects translate into quantifiable functional improvements [57]. Investigating the interplay between these psychological benefits and physiological pain mechanisms could pave the way for more integrative and effective pain management strategies. In addition, the long-lasting impact of psilocybin raises concerns about dose optimization and the potential for tolerance or adverse psychological effects with repetitive use [58].

As a powerful hallucinogen, psilocybin can induce intense psychological experiences that, while potentially therapeutic in controlled settings, could be destabilizing or even traumatic for some individuals [59]. However, despite concerns about the potential for abuse and addiction, current evidence suggests that psilocybin has a low potential for physical dependence compared to many other substances and a low, almost negligible, incidence of adverse events within the therapeutic dosing intervals used for pain control [60].

Additionally, worries about how different patients might react due to individual genetic, brain biology, or mental health factors mean that it is necessary to focus on personalized treatment plans that provide the best results with the least risk [61].

Complementing our emphasis on personalized treatment plans, a potential limitation that warrants targeted clinical testing is sex-related heterogeneity. In Long–Evans rats, psilocybin (1 mg/kg, oral) elicited robust 5-HT2A–mediated behavioral responses in adults but not adolescents, with adult females > males; within females, responses were higher in diestrus than proestrus, indicating modulation by hormonal state and aligning with prior evidence that estradiol can regulate 5-HT2A signaling [62]. To our knowledge, current clinical studies in pain have not prospectively stratified outcomes by sex or hormonal status. Future trials should therefore pre-plan sex-disaggregated analyses, capture endocrine variables (e.g., menstrual phase, menopause, hormone therapy), and consider potential dose/response tailoring to minimize risk while maximizing benefit.

### 3.3. Ethical Considerations

The ethical implications of using a potent psychoactive compound for emotional modulation warrant attention. Research on psychedelics requires the development of informed consent procedures that are both robust and comprehensive. Informed consent mechanisms must go beyond traditional frameworks to account for the unique nature of psychedelic interventions, anticipating rare but severe complications, such as seizures or psychotic episodes as well as panic attacks or mood instability [63]. For instance, participants may have limited understanding of altered states of consciousness during the psychological phenomenon of ego dissolution, a hallmark of many psychedelic experiences, or the risks associated with such experiences, i.e., disorientation or distress, necessitating innovative methods such as multimedia formats or preparatory workshops to enhance comprehension [64].

Screening participants rigorously to exclude high-risk individuals such as those with a history of psychosis, seizure disorders, or severe mood instability is critical for ethical safety. Over-restrictive protocols may lead to the exclusion of individuals who could benefit greatly from psychedelic therapies, particularly in cases where standard treatments are ineffective. Personalized risk assessments can be informed by factors such as participants’ mental health histories, genetic predispositions, and current psychosocial contexts. However, integrating these considerations into study designs requires significant resource allocation and innovative methodologies [65].

Adequate preparation for participants is a foundational aspect of conducting psychedelic research ethically. In our opinion, preparatory measures must go beyond generic guidelines to account for individual differences in psychological readiness. Tailored approaches, including one-on-one sessions with trained therapists or guided visualization exercises, may help participants anticipate and navigate the psychedelic experience more effectively. In addition, researchers must also prepare participants for the variability in psychedelic outcomes, ranging from profound emotional breakthroughs to challenging psychological moments, by equipping them with coping mechanisms [66]. Measures such as ensuring a comfortable setting, having emergency medical support on standby, and providing post-treatment psychological integration sessions are crucial.

Risk management strategies for conducting ethical studies involving psychedelics must extend beyond acute interventions to include longer-term follow-ups. Addressing delayed side effects or psychological harm that may emerge after treatment sessions is critical [67].

Patients with chronic pain often experience heightened societal stigma and marginalization, a vulnerability that can be compounded by co-occurring substance use disorders or mental health challenges, such as depression or suicidal ideation [7]. Ethical research must actively address these vulnerabilities to create inclusive and supportive environments for participants. However, active monitoring of the potential risk of abuse of these substances should be ensured, preventing the risk through education and psychological supervision and discontinuing treatment at the slightest sign of misuse.

The question of whether psilocybin could lead to a new addiction crisis is complex. While psilocybin itself is not considered highly addictive, there are concerns about psychological dependence and the potential for its use to act as a gateway to other, more dangerous substances. Additionally, the normalization of psychedelic use could potentially lead to increased recreational use and associated risks.

The establishment of specialized review committees dedicated to psychedelic research could balance the need for efficiency with the demand for comprehensive safety assessments. Furthermore, transparent communication about the scientific basis, benefits, and safeguards related to psychedelic therapies is critical to promoting public trust [64]. Indeed, overlooking or under-reporting negative effects to emphasize positive findings could harm public confidence and hinder the possible benefits of psychedelics in clinical settings. Equally important is addressing society’s concerns about the potential non-clinical misuse of psychedelic substances through rigorous regulatory frameworks.

Aside from the specific context of psilocybin, equitable access is a fundamental ethical responsibility in medicine that necessitates deliberate attention during the formulation and implementation of any innovative treatment. Ethical interpretations and regulatory approaches differ significantly among countries and health systems: principles such as beneficence, non-maleficence, autonomy, and justice may be prioritized differently, resulting in diverse thresholds for risk-benefit assessment, informed consent mandates, and protections for vulnerable groups [68]. It is crucial to consider these factors to guarantee that psilocybin-assisted therapy, if validated as effective, remains accessible to all, rather than being limited to individuals with greater resources or in regions with more lenient ethical standards, thereby promoting equitable and proportional access for chronic pain populations.

## 4. Conclusions

Psilocybin is emerging as a promising therapeutic candidate in the context of chronic pain, acting through mechanisms that include neuroplasticity enhancement, serotonergic modulation, and anti-inflammatory effects. Early preclinical studies consistently demonstrate durable analgesic and anxiolytic effects, while clinical investigations suggest improvements in mood, resilience, and, in some cases, pain perception. These findings highlight psilocybin’s ability to address both the sensory and affective dimensions of pain, offering a multidimensional approach that differs from traditional analgesics. Nevertheless, evidence remains preliminary, with few randomized controlled trials and small sample sizes. Safety concerns, ethical issues, and regulatory challenges further underline the need for rigorous study designs and standardized protocols. Future research should prioritize dose optimization, long-term follow-up, and integration within multidisciplinary pain management strategies. Taken together, psilocybin represents not a replacement but a potential complement to existing treatments, providing new perspectives for the management of complex and refractory pain conditions.

## Figures and Tables

**Table 1:** Clinical evidence on the use of psilocybin for pain.

Study	Population	Dose	Design	Follow Up	Outcomes	Adverse Effect
Griffiths [47]	Advanced cancer (*n* = 51)	22 or 30 mg/70 kg psilocybin	RCT vs. placebo like (1 or 3 mg/70 kg)	6 months	Sustained improvement in mood, quality of life, optimism.	Rare, generally well tolerated
Lyes [50]	Chronic pain patients (*n* = 3)	250–1000 mushroom powder	Observational	Over 6 months in 1 case	Reported analgesic effect with improved function	None reported
Schindler [51]	Adults with migraine (*n* = 10)	0.143 mg/kg	RCT crossover vs. placebo	2 weeks	Reduction in weekly migraine days	None reported
Schindler [52]	Cluster headache (*n* = 14)	0.143 mg/kg, 3 doses once every 5 days	RCT vs. placebo	8 weeks	No statistical difference in frequency, duration, or intensity of attacks	None reported
Schindler [53]	Cluster headache (continuation of [51]) (*n* = 10)	0.143 mg/kg, 3 doses once every 5 days, second cycle after at least 6 months	Observational	8 weeks	Significant reduction in cluster attack frequency	None reported
Rucker [54]	SUNHA (*n* = 4)	5 mg on day 1, 7.5 mg on day 6, and 10 mg on day 11	Observational	39 days	Two participants had a >50% improvement in headache frequency	None reported
Aday [13]	Adults with fibromyalgia (*n* = 5)	15 mg and 25 mg 2 weeks apart	Observational		2.3 ± 1.3-point decrease in pain severity; 9.4 ± 4.2-point decrease in pain interference; 2 ± 2.8-point increase in chronic pain acceptance	Mild, transient psychological discomfort
Jevotovsky [55]	Complex Regional Pain Syndrome (*n* = 1)	2 g on day 1, 5.5 g on day 3, and 3.5 g on day 5; *Psilocybe cubensis* mushrooms	Case report	9 months	From NRS 4 to NRS 0–1	None reported

Abbreviations: RCT, Randomized Controlled Trial; NRS, Numeric Rating Scale.

## Data Availability

No new data were created or analyzed in this study.

## Supplementary Materials

The following supporting information can be downloaded at: https://www.mdpi.com/article/10.3390/medsci13040277/s1, Table S1: Exact database-specific search queries.
