# Supplementary material for: Psilocybin and Chronic Pain: A New Perspective for Future Pain Therapists?
Source: Med Sci (Basel). 2025 Nov 20;13(4):277. doi: 10.3390/medsci13040277 (PMC12641700; doi:10.3390/medsci13040277)
Supplement: Supplementary file 1 [file medsci-13-00277-s001.zip › medsci-3942207-supplementary.pdf]

Supplementary Table S1. Exact database-specific search queries (Updated)

A comprehensive literature search was conducted in PubMed/MEDLINE, Google Scholar, and Web of Science, covering the period from January 2000 to March 2025. Only studies published in English were considered. The exact, database-specific strings and applied limits are reported below.

Database	Query Type	Exact Search Query	Notes / Limits
PubMed/MEDLINE	Preclinical	(("Psilocybin"[Mesh] OR psilocybin[Title/Abstract]) AND (pain[Title/Abstract] OR nociception[Title/Abstract] OR neuropathic[Title/Abstract] OR inflammatory[Title/Abstract] OR mechanism[Title/Abstract] OR neuroplasticity[Title/Abstract] OR neuroinflammation[Title/Abstract])) AND (("Animals"[Mesh] NOT "Humans"[Mesh]) OR "Cell Line"[Mesh] OR "In Vitro Techniques"[Mesh]) AND (english[lang]) AND ("2000/01/01"[Date - Publication] : "2025/03/31"[Date - Publication])	Filters embedded in query: English language and publication date range 2000-01-01 to 2025-03-31.
PubMed/MEDLINE	Clinical	(("Psilocybin"[Mesh] OR psilocybin[Title/Abstract]) AND ("chronic pain"[Title/Abstract] OR "neuropathic pain"[Title/Abstract] OR "nociplastic pain"[Title/Abstract] OR headache[Title/Abstract] OR migraine[Title/Abstract] OR "cluster	Filters embedded in query: Humans[Mesh], English language, publication date range 2000-01-01 to 2025-03-31.

		headache"[Title/Abstract] OR fibromyalgia[Title/Abstract] OR "complex regional pain syndrome"[Title/Abstract] OR "Migraine Disorders"[Mesh] OR "Fibromyalgia"[Mesh] OR "Complex Regional Pain Syndromes"[Mesh] OR "Headache Disorders"[Mesh])) AND "Humans"[Mesh] AND (english[lang]) AND ("2000/01/01"[Date - Publication] : "2025/03/31"[Date - Publication])	
Google Scholar	Preclinical	psilocybin AND (pain OR nociception OR neuropathic OR inflammatory OR mechanism OR neuroplasticity OR neuroinflammation)	Apply UI filters: Custom range 2000–2025 (screened through March 2025) and Language: English only. Document types: Articles/Reviews where applicable.
Google Scholar	Clinical	psilocybin AND ("chronic pain" OR "neuropathic pain" OR "nociplastic pain" OR headache OR migraine OR "cluster headache" OR fibromyalgia OR "complex regional pain syndrome")	Apply UI filters: Custom range 2000–2025 (screened through March 2025) and Language: English only. Use 'Sort by relevance' and screen titles/abstract snippets for preclinical/clinic

			al distinction.
Web of Science Core Collection	Preclinical	`TS=(psilocybin AND (pain OR nociception OR neuropathic OR inflammatory OR mechanism OR neuroplasticity OR neuroinflammation)) AND LA=(English) AND PY=(2000-2025)`	Advanced Search. Timespan set to 2000–2025 (records screened through March 2025). Language filter: English. Specify indexes searched (e.g., SCI-EXPANDED, ESCI) in the Methods if needed.
Web of Science Core Collection	Clinical	`TS=(psilocybin AND ("chronic pain" OR "neuropathic pain" OR "nociplastic pain" OR headache OR migraine OR "cluster headache" OR fibromyalgia OR "complex regional pain syndrome")) AND LA=(English) AND PY=(2000-2025)`	Advanced Search. Timespan set to 2000–2025 (records screened through March 2025). Language filter: English. Index selection as per Methods; preclinical/clinical nature confirmed at screening.

Note: Some platforms limit date filters to the year level (e.g., Google Scholar, Web of Science). Where needed, records within 2025 were screened to include items published up to March 2025 only.
